# The effects of Tai Chi on executive function and neurophysiological outcomes in college students: a systematic review and meta-analysis

**DOI:** 10.3389/fpsyg.2026.1955322

**Published:** 2026-10-15

**Authors:** Darrin Sime Nkemeni, Hao Hong

**Affiliations:** Wushu College of Henan University, Henan University, Kaifeng, China

**Keywords:** cognitive flexibility, college students, executive function, inhibitory control, mind–body exercise, Tai Chi, working memory

## Abstract

**Background:**

College students face substantial cognitive demands, motivating scalable non-pharmacological approaches to support executive function. Tai Chi combines low-to-moderate physical activity, motor-sequence learning, postural control, breathing regulation, and sustained attention, but evidence in young adults remains uncertain.

**Objective:**

To evaluate the effects of Tai Chi on objective executive-function outcomes and to summarize associated neurophysiological findings in university students.

**Methods:**

Following PRISMA 2020, PubMed/MEDLINE, Web of Science Core Collection, Scopus, and CNKI were searched from inception to 15 April 2026, with an English-language restriction. Eligible studies evaluated structured Tai Chi against a concurrent comparator and reported objective executive-function and/or prespecified neurophysiological outcomes. Two reviewers screened records, extracted data, and assessed randomized trials using RoB 2. Certainty was evaluated using GRADE. Hedges’ g was pooled using DerSimonian–Laird random-effects models; REML and Hartung–Knapp analyses assessed robustness. The present review was not prospectively registered; PROSPERO CRD420261480624 concerns a future update.

**Results:**

Seven randomized trials were eligible (*n* = 425), and four comparisons entered the primary selected-task meta-analysis (*n* = 248). The pooled estimate favored Tai Chi (*g* = 1.05, 95% CI 0.23–1.87; *i*^2^ = 89.0%) but was dominated by one task-congruent working-memory trial and was not robust under Hartung–Knapp inference (95% CI − 0.56 to 2.67; *p* = 0.130). Inhibitory-control performance improved across three trials (*n* = 193; g = 0.57, 95% CI 0.28–0.85; *i*^2^ = 0%), although certainty was low. Working memory effects were nonsignificant for the primary selected metric (*g* = 1.00, 95% CI − 0.37 to 2.37) and correct responses/accuracy (*g* = 0.79, 95% CI − 0.19 to 1.77). Cognitive flexibility was represented by one trial (*g* = 0.29, 95% CI − 0.20 to 0.77). Neurophysiological findings were exploratory. No study reported objective academic outcomes.

**Conclusion:**

Tai Chi may improve short-term inhibitory-control performance in college students, but evidence for broader executive-function benefits is insufficient. Larger preregistered trials using active comparators, task-neutral measures, longer follow-up, and objective academic outcomes are required.

## 1 Introduction

Executive functions (EFs) denote a set of top-down cognitive processes that support goal-directed behaviors, self-regulation, and adaptive decision-making, particularly in situations where reliance on automatic or habitual responses would be insufficient (Diamond, 2013). According to the widely adopted framework of Miyake et al. (2000), subsequently refined by Diamond (2013), EFs comprise three core, partially dissociable components: inhibitory control, defined as the capacity to suppress prepotent responses and resist interference; working memory, the ability to maintain and manipulate information over brief intervals; and cognitive flexibility, the capacity to shift between mental sets or task rules. These core processes constitute the foundation of higher-order cognition—including reasoning, planning, and problem-solving—and are robust predictors of academic attainment and socio-emotional adjustment (Diamond, 2013).

The university years represent a developmental period in which the demands placed upon executive control are particularly pronounced. Undergraduate and postgraduate students contend with substantial cognitive workloads under conditions of elevated psychological stress, irregular sleep patterns, and considerable lifestyle transition (Guo et al., 2022). Inefficiencies in executive control during this period have been associated with academic procrastination, poor time management, emotional dysregulation, and maladaptive behavioral patterns, most notably excessive screen exposure and problematic smartphone use (Li et al., 2025a); deficits in inhibitory control have been implicated in mobile phone addiction among college students (Li et al., 2025b). Physical exercise is well established as an efficacious non-pharmacological approach to cognitive enhancement (Lom, 2017), and recent meta-analytic evidence indicates that even acute bouts of physical activity confer measurable benefits for working memory and inhibition in young adults (Garrett et al., 2024). Nevertheless, conventional unimodal aerobic and resistance training regimens principally load the cardiovascular and musculoskeletal systems and impose comparatively limited demands on complex motor coordination or attentional networks (Srinivas et al., 2021).

Tai Chi, a mind–body exercise derived from traditional Chinese martial arts, may address this limitation. The practice integrates low-to-moderate-intensity physical activity with slow, deliberate motor sequencing, continuous dynamic balance regulation, diaphragmatic breathing, and sustained attentional engagement (Huston and McFarlane, 2016; Chen et al., 2021). In contrast to largely automatized aerobic modalities such as treadmill running or stationary cycling, Tai Chi constitutes a continuous cognitive–motor dual task (Wu et al., 2021): practitioners must retain and retrieve extended movement sequences (working memory), regulate posture and centre of gravity whilst suppressing extraneous movement (inhibitory control), and alternate attention between interoceptive signals and the external environment (cognitive flexibility) (Diamond, 2013; Wu et al., 2021). On theoretical grounds, therefore, Tai Chi possesses characteristics that render it a plausible candidate for the targeted enhancement of executive function.

The empirical evidence, however, derives predominantly from populations that differ substantially from young adults. Meta-analytic syntheses have documented significant cognitive benefits of Tai Chi in cognitively healthy older adults—Wayne et al. (2014) reported a large effect on executive function relative to non-active controls (Hedges’ *g* = 0.90)—and in clinical cohorts with mild cognitive impairment (Wu et al., 2013; Chen et al., 2024; Yang et al., 2020). Extrapolation of these findings to university students is problematic: whereas older adults typically exhibit age-related cognitive decline, young adults perform at or near peak cognitive capacity and present distinct neurobiological baselines, potentially attenuating the magnitude of detectable training effects (Wang and Lyu, 2024). Moreover, the limited number of trials conducted in tertiary student populations has yielded inconsistent findings. A twelve-week randomized controlled trial employing electroencephalography reported improved accuracy and reduced reaction time on the Stroop task following 24-form simplified Tai Chi training (Wang and Lyu, 2024), whereas an eight-week mindfulness-based Tai Chi intervention produced significant improvements in inhibition and shifting but no reliable effect on working memory updating (Li et al., 2025b). Interpretation of this literature is further complicated by marked methodological heterogeneity across trials, encompassing differences in Tai Chi style (e.g., 24-form Simplified versus Yang-style), intervention dose and duration, the nature of comparator conditions, and the neurophysiological recording modalities employed, including resting-state EEG, event-related potentials, and functional near-infrared spectroscopy (Wang and Lyu, 2024; Wang et al., 2025a; Wang et al., 2025b).

Two methodological issues are particularly important in this population. First, young adults may perform near ceiling on simple cognitive tasks, reducing sensitivity to intervention-related change. Second, passive control groups cannot distinguish effects specific to Tai Chi from nonspecific effects of structured physical activity, instructor attention, expectancy, and social interaction. These limitations require cautious interpretation of effect magnitude and generalizability.

Interpretation also depends on whether outcomes represent near transfer to training-related stimuli or broader transfer to task-neutral executive-function measures. In addition, inhibitory control, working-memory updating, cognitive flexibility, sustained attention, and emotional-attentional processing should not be treated as interchangeable constructs. Neurophysiological outcomes are therefore considered exploratory and synthesized narratively because the available fNIRS, ERP, and resting-state EEG measures assess different processes and are not sufficiently homogeneous for quantitative pooling.

To address these ambiguities, this systematic review and meta-analysis aims to:

1. Synthesize available evidence on the effects of Tai Chi on core executive function subdomains in college students.
2. Formally quantify pooled effect sizes using random-effects meta-analytic models and conduct sensitivity analyses to identify sources of heterogeneity.
3. Systematically evaluate secondary neurophysiological and electrophysiological correlates and physical-fitness outcomes.
4. Assess whether laboratory-measured executive improvements transfer to real-world behavioral self-control or objective academic achievement metrics.

## 2 Methods

### 2.1 Protocol and registration

This systematic review and meta-analysis was conducted and reported in strict compliance with the Preferred Reporting Items for Systematic Reviews and Meta-Analyses (PRISMA 2020) guidelines (Parums, 2021). The present review was not prospectively registered, and no dated public protocol preceded screening or analysis. This absence is retained as a limitation. PROSPERO CRD420261480624, registered on 18 August 2026, applies only to a future update and is not presented as prospective registration of the current review. During the eligibility and data audit, records were reassessed against the stated PICOS criteria. Protocols, cross-sectional exposure comparisons, trials without confirmed student enrolment, studies without Tai Chi exposure, and multicomponent interventions from which the Tai Chi effect could not be isolated were excluded from the intervention evidence base (Figure 1).

**Figure 1:**
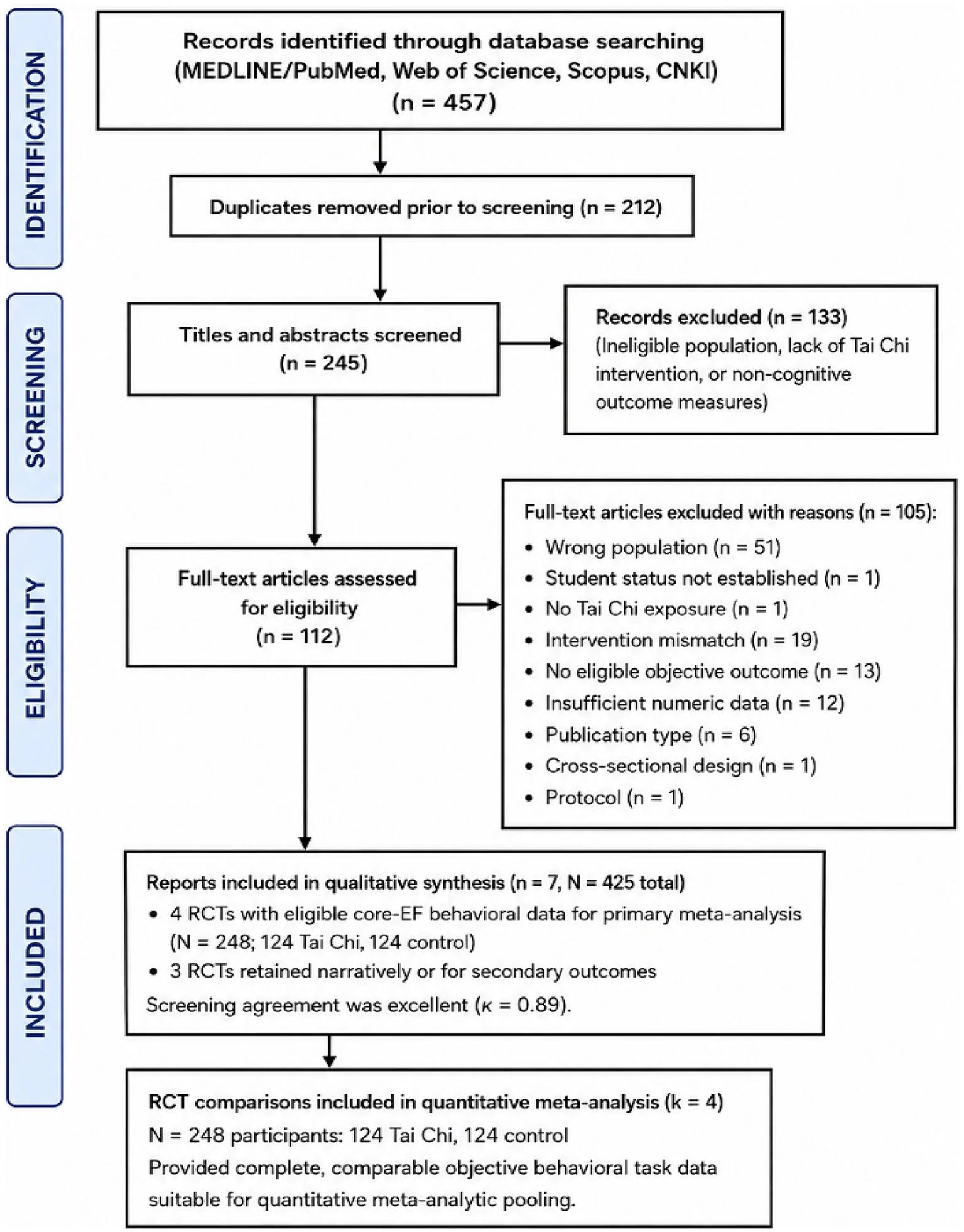
PRISMA representation.

### 2.2 Eligibility criteria

*Population*: Participants were currently enrolled in undergraduate, graduate, or other tertiary-education programs, generally with a mean age of 18–25 years. Mixed samples were eligible only when student data were separable. General young-adult samples without explicit student enrolment were excluded.

*Intervention/exposure*: Tai Chi of any recognized style had to be delivered as a structured and isolable intervention. Multicomponent interventions were excluded when the independent Tai Chi contribution could not be estimated and Tai Chi was not the primary component. Observational exposure studies were excluded because they could not estimate intervention effects.

*Comparator*: Eligible studies required a concurrent passive or active comparison group.

*Outcomes*: Studies were eligible if they reported at least one objective behavioral EF outcome (inhibitory control, working-memory updating, or cognitive flexibility) or a prespecified neurophysiological outcome relevant to executive control (fNIRS oxygenated hemoglobin, ERP N2/P3, or resting-state EEG spectral power). Self-report and fitness outcomes were secondary and could not establish eligibility by themselves. Objective academic outcomes included GPA, examination scores, course completion, or retention.

*Study design*: Completed randomized and non-randomized comparative intervention studies were eligible. Protocols, narrative reviews, editorials, qualitative studies, and uncontrolled single-arm studies were excluded. Quantitative synthesis was limited to RCTs with extractable between-group behavioral data.

The restriction to intervention studies was adopted during the revised eligibility audit to align the evidence base with the causal wording of the review question. Cross-sectional comparisons of experienced practitioners can identify associations and generate hypotheses, but they cannot separate practice effects from self-selection, prior fitness, socioeconomic background, motivation, or other stable group differences. Similarly, protocols identify forthcoming research but contain no effect estimate and cannot contribute evidence about intervention efficacy. Excluding both record types produces a narrower but conceptually coherent evidence base.

Eligibility based on neurophysiological outcomes was retained even when a trial did not report an objective EF task, provided that the neural measure had been prespecified as relevant to executive control. Such trials inform the secondary narrative question but not the behavioral meta-analysis. Fitness or self-report outcomes alone were insufficient for inclusion because the review was not designed as a general review of Tai Chi and student health (Table 1).

**Table 1:** Eligibility criteria (PICOS framework).

Parameter	Inclusion criteria	Exclusion criteria
Population (P)	Enrolled undergraduate or postgraduate students (mean age 18–25 years)	Clinical populations with major neurological/psychiatric conditions; non-college cohorts without disaggregated data
Intervention (I)	Tai Chi (any style: 24-form, Yang-style, MTCC, Bafa Wubu) as primary intervention component	Interventions where Tai Chi cannot be disaggregated from unimodal physical activities
Comparator (C)	Concurrent control group (wait-list, passive control, active exercise, cognitive training)	No control group (uncontrolled single-arm trials excluded from quantitative meta-analysis)
Outcomes (O)	Primary: Objective behavioral tasks measuring IC, WM, or CF. Secondary: fNIRS, EEG, ERP, self-control scales, fitness	Exclusively self-reported executive function without objective task-based outcomes
Study design (S)	Completed randomized or non-randomized comparative intervention study; pooling limited to eligible RCTs	Cross-sectional exposure study, protocol, review, commentary, qualitative or uncontrolled study

### 2.3 Information sources and search strategy

PubMed, Web of Science Core Collection, Scopus, and CNKI were searched from database inception to 15 April 2026, with an English-language restriction and no date or publication-status restrictions. The search strategy combined MeSH terms and free-text terms using Boolean operators: (“Tai Ji” OR “Tai Chi” OR “Tai Ji Quan” OR “Tai Chi Chuan” OR “Taijiquan”) AND (“college student” OR “university student” OR “undergraduate” OR “postgraduate” OR “young adult”) AND (“executive function” OR “executive control” OR “inhibitory control” OR “inhibition” OR “working memory” OR “updating” OR “cognitive flexibility” OR “task switching” OR “Stroop” OR “Flanker” OR “Go/No-Go” OR “n-back”). Reference lists of retrieved articles and relevant reviews were manually searched to identify additional studies. Exact interface-specific strategies are reproduced in Appendix A.

### 2.4 Study selection and data extraction

All retrieved records were exported to EndNote 20 for automated and manual deduplication. Two reviewers independently screened titles and abstracts against eligibility criteria. Full texts of potentially eligible articles were reviewed independently. Discrepancies were resolved through consensus or adjudication by a senior third reviewer. Inter-reviewer reliability was calculated using Cohen’s kappa (κ). Data were extracted using a standardized data extraction template capturing:

- Publication details (first author, publication year, country, trial design)
- Participant demographics (sample size, sex distribution, mean age, health/behavioral status)
- Intervention characteristics (style, duration in weeks, frequency, session length, intensity)
- Comparator details
- Cognitive task metrics (means and standard deviations for pre/post scores or change scores for reaction time and accuracy)
- Secondary neuroimaging, EEG, ERP, or fitness parameter

Potential overlapping reports were assessed by comparing authorship, recruitment setting, country, registration, sample characteristics, intervention dose, and outcome battery. Wang and Lyu (2024) and Wang et al. (2025a) were treated as independent because they involved different author groups and countries and reported non-overlapping primary outcomes (Figure 2).

**Figure 2:**
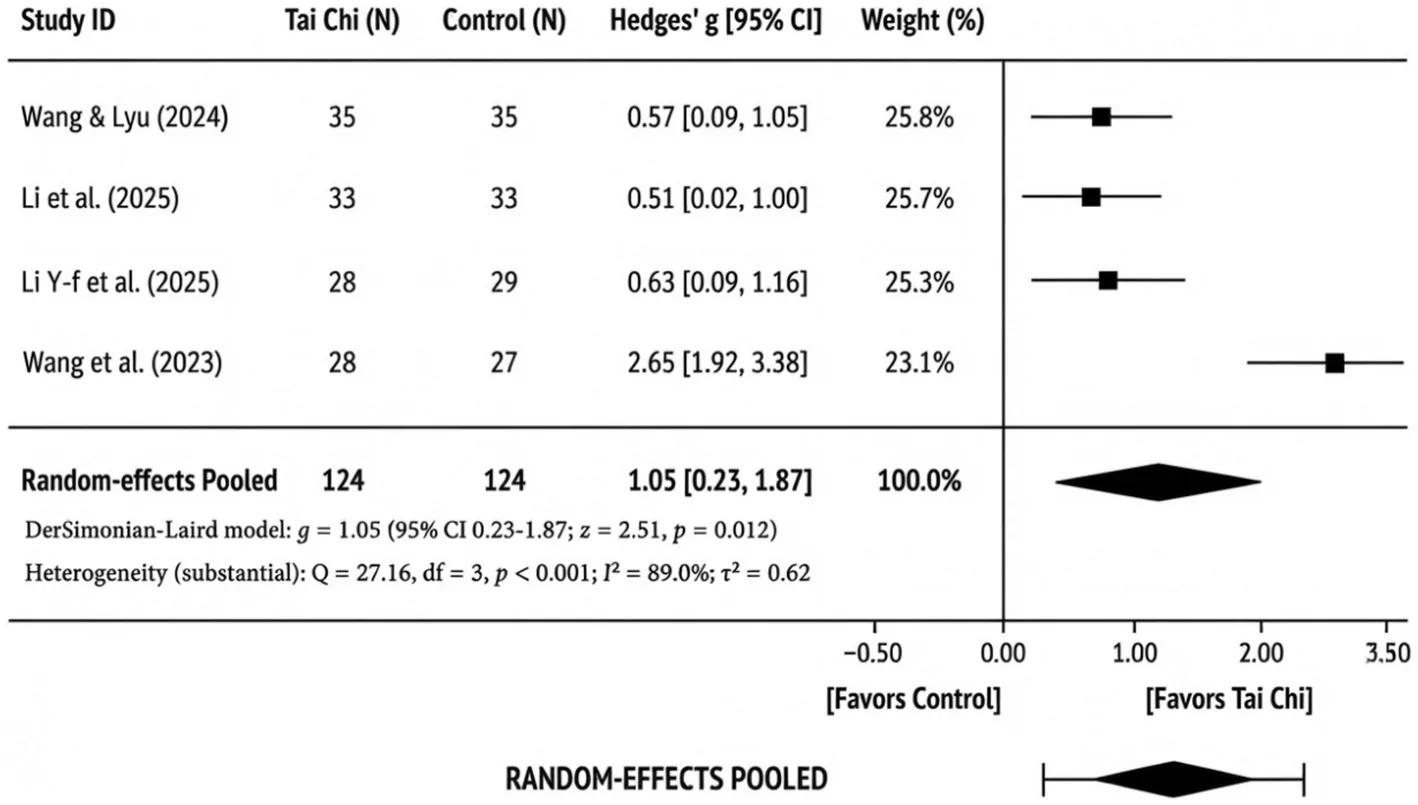
Overall selected-task model.

The complete extraction contained 32 behavioral rows. Group sizes, means, SDs, task conditions, time points, and effect directions were independently cross-checked. Wang and Lyu (2024) reaction times were converted from seconds to milliseconds by multiplying both means and SDs by 1,000; this linear conversion did not alter Hedges’ g.

Tongtong (2022) was classified as qualitative inhibitory-control and fNIRS evidence but was not pooled because the report did not provide the control-group means and SDs required to calculate a concurrent-control behavioral effect.

#### 2.4.1 Outcome selection, time points, and unit-of-analysis rules

1. One comparison per trial entered the overall selected-task model. Interference/incongruent conditions were prioritized; reaction time was prioritized over accuracy when both were available; immediate post-intervention assessment was used; and post-test group means/SDs were used when adjusted effects or change-score variances were unavailable.
2. One effect per trial per subdomain entered each subdomain model. No study appeared more than once within a single model.
3. For Li et al. (2025c), only the Tai Chi and overuse-control arms were compared; the low-use reference arm was not pooled.
4. All effects were oriented so that positive Hedges’ g indicated better Tai Chi performance—shorter reaction time or higher accuracy/score.
5. Working memory was analyzed using a primary selected-metric model—reaction time prioritized, with the digit-string score used where RT was unavailable—and an exploratory correct-response/accuracy model. The two models were separate and never combined.
6. Cognitive flexibility was represented by one study and was not meta-analyzed. Hao and Yuan behavioral findings were retained narratively for the reasons stated above.

These rules were made explicit during revision and were not documented in a prospectively registered analysis plan. They are therefore reported as revised-analysis rules rather than described as prospectively prespecified (Figure 3).

**Figure 3:**
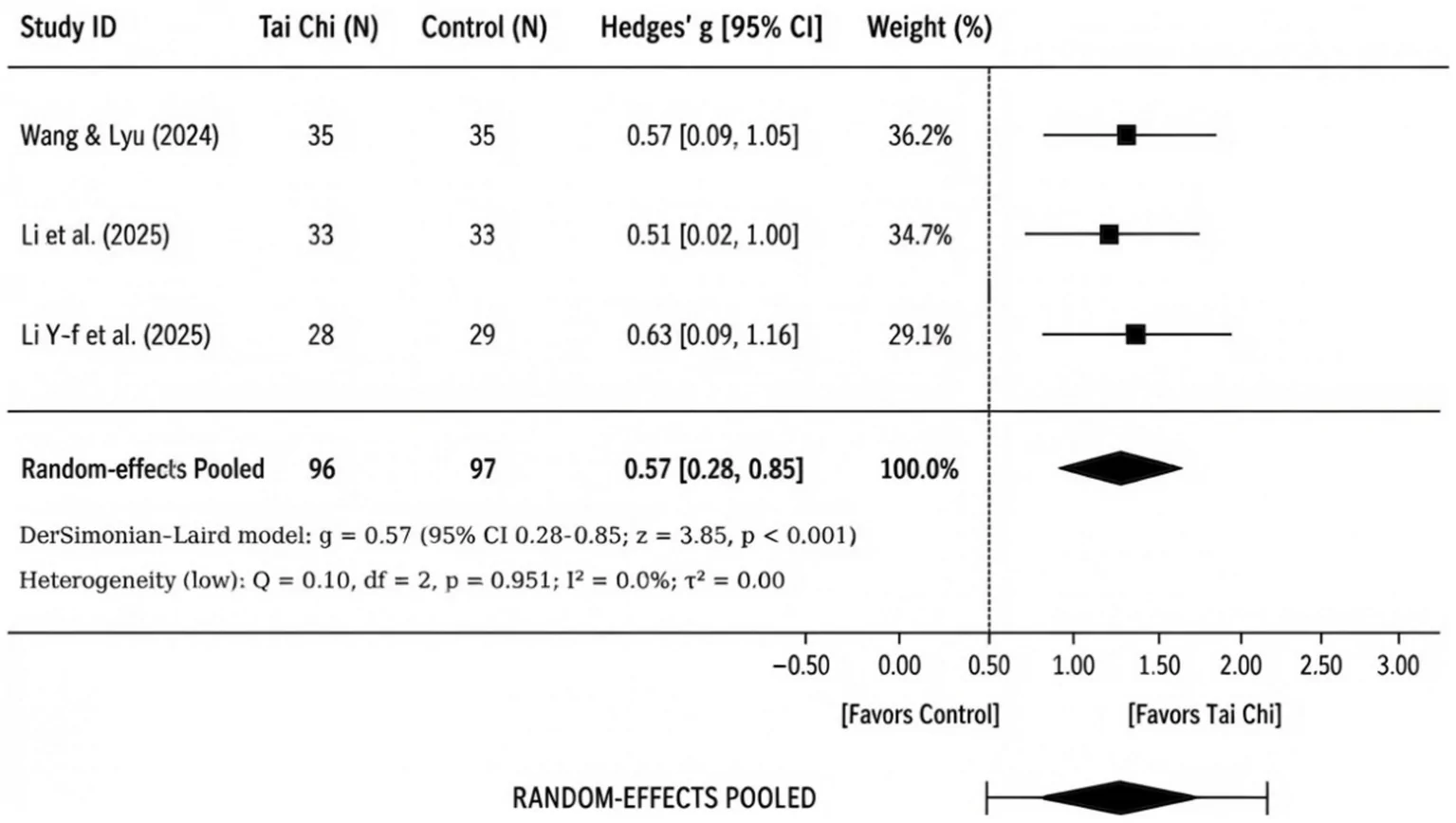
Inhibitory-control model.

### 2.5 Risk of Bias assessment

RCTs were assessed using RoB 2 (Sterne et al., 2019). ROBINS-I was planned for eligible non-randomized intervention studies (Sterne et al., 2016), but none remained after the final eligibility audit. Certainty of evidence was assessed using GRADE (Balshem et al., 2011).

### 2.6 Statistical analysis

Quantitative meta-analysis was performed using R software (version 4.3.2, packages meta and metafor) and RevMan (version 5.4). Standardized mean differences (Hedges’ g) with 95% confidence intervals (CIs) were computed for continuous behavioral outcomes (reaction times and accuracy rates). Where studies reported both reaction time and accuracy rates, reaction time (RT) was prioritized as the primary standardized metric, harmonized such that positive effect sizes (*g* > 0) consistently indicate superior executive performance (e.g., shorter reaction times or higher accuracy) (Hedges, 1982).

A DerSimonian–Laird random-effects model (DerSimonian and Laird, 1986) was used as the primary estimator. Between-study heterogeneity was evaluated using Cochran’s *Q* test (significance at *p* < 0.10) and quantified via the *i*^2^ statistic (25% low, 50% moderate, 75% high heterogeneity). Subdomain analyses were conducted across EF subdomains: inhibitory control, working-memory updating, and cognitive flexibility.

Sensitivity analyses were conducted using leave-one-out cross-validation and by excluding trials utilizing task-congruent cognitive paradigms. Formal tests of funnel-plot asymmetry and trim-and-fill were not conducted because only four comparisons entered the primary model. For each independent-group comparison, the oriented mean difference was divided by the pooled within-group SD and multiplied by the small-sample correction factor to obtain Hedges’ g. Sampling variances incorporated group sizes and effect magnitude. Generic inverse-variance input files containing yi and vi were retained for reproducibility.

REML and Hartung–Knapp–Sidik–Jonkman inference were used as sensitivity analyses because conventional normal-theory intervals may be overconfident with very few studies. The overall result was interpreted as non-robust when Hartung–Knapp inference crossed the null.

A conventional independent-effects *Q*-between test was not interpreted because Li et al. (2025b) and Li et al. (2025c) contributed outcomes to both inhibitory-control and working-memory models, making the estimates dependent. Within-study covariance was unavailable, and only four unique trials contributed across the two multi-study domains, which was insufficient for reliable cluster-robust or multilevel moderator inference.

Formal funnel-plot asymmetry tests and trim-and-fill were omitted after the final audit because only four comparisons entered the primary model. Publication bias and small-study effects could not be assessed reliably.

Forest plots were restricted to syntheses involving at least two studies. They display group sizes, study effects and confidence intervals, random-effects weights, pooled estimates, *Q*, *i*^2^, and *τ*^2^. The one-study cognitive-flexibility result was reported in text and tables without a pooled diamond or heterogeneity statistic.

## 3 Results

### 3.1 Study selection

The searches identified 457 records. After 212 duplicates were removed, 245 records were screened and 133 were excluded at title/abstract stage. Of 112 full-text reports assessed, 105 were excluded. Seven completed RCT reports were included, totaling *n* = 425 participants. Four RCT comparisons (*n* = 248; 124 Tai Chi and 124 control) provided eligible core-EF behavioral data and entered the primary selected-task meta-analysis. Three RCTs were retained only narratively or for secondary outcomes: Yuan et al. (2025) contributed affective oddball/ERP evidence but no behavioral measure of a core EF domain, Wang et al. (2025a) reported an eligible neurophysiological outcome but no objective EF task, and Tongtong (2022) did not provide sufficient controlled summary statistics for effect-size calculation. The Tai Chi–jogging–HIIT study (Sun et al., 2025) was excluded because Tai Chi was not isolable and was not the primary intervention component. Screening agreement was excellent (*κ* = 0.89; Table 2).

**Table 2:** Full-text exclusions.

Exclusion category	Reason	*n*
Wrong population	Non-college, clinical, or inseparable mixed samples	51
Student status not established	Cui et al. (2021): paid healthy adults not identified as students	1
No Tai Chi exposure	Zhu et al. (2023)	1
Intervention mismatch	Tai Chi not isolable/not primary, including Sun et al. (2025)	19
No eligible objective outcome	Only self-report or ineligible outcomes	13
Insufficient numeric data	Required sample sizes/dispersion/comparative statistics unavailable	12
Publication type	Review, commentary, or qualitative article	6
Cross-sectional design	Darrin et al. (2025): exposure comparison, not an intervention study	1
Protocol	Rodríguez-Vera et al. (2026): no empirical results	1
Total		105

### 3.2 Included study characteristics

The seven included RCTs enrolled *n* = 425 participants. Six were conducted in China and one in Thailand. Mean ages were approximately 18.6–21.0 years. Tai Chi formats included 24-form Simplified Tai Chi, simplified Yang-style, mindfulness-based Tai Chi and unspecified Taijiquan. RCTs generally lasted 8–15 weeks, with 3 sessions per week and 40–60 min per session where reported. All four comparisons in the primary selected-task model used passive or routine-activity controls; no pooled comparison used an active exercise comparator.

Core-EF behavioral tasks assessed Stroop, Flanker, n-back/updating, digit-string memory, and More-Odd shifting performance. Go reaction time in Li et al. (2025c) was classified by the primary article as sustained attention and was not pooled as inhibition. The Yuan et al. affective oddball task was classified as emotional-attentional processing rather than a core EF measure. Prespecified neurophysiological outcomes included fNIRS, ERP, and resting-state EEG. No eligible study reported objective GPA, examination, retention, or course-completion outcomes.

Wang and Lyu randomized 70 students to 12 weeks of 24-form Tai Chi or routine activity and reported post-intervention differences on incongruent Stroop reaction time, alongside frontal resting EEG measurements. The trial was directly relevant to inhibitory control but contained internal reporting inconsistencies regarding recruitment and descriptive sample information. Li et al. (2025b) randomized 66 male students to an eight-week mindfulness-based Tai Chi program or routine activity and administered inhibition, updating, and shifting tasks. Its multiple outcomes made it particularly important to apply the one-effect-per-model rule.

Li et al. (2025c) studied students characterized by short-video overuse in a three-arm design. Only the Tai Chi and overuse-control arms were used for the intervention contrast; the low-use observational reference arm was excluded from pooling. Verification of the article showed that Go reaction time was interpreted as sustained attention, not response inhibition. The revised inhibitory-control extraction therefore uses the Stroop post-test contrast, while the digit-string score contributes separately to the working-memory model. Follow-up findings suggested attenuation after training stopped, but follow-up was not pooled because the review selected immediate post-intervention assessment.

Yuan et al. studied 40 female students using an affective oddball categorization paradigm and ERP recording. Participants responded to both standard and deviant emotional images; the task measured emotional-attentional processing rather than suppression of a prepotent response. Its behavioral reaction time and N2/P3 findings were therefore synthesized narratively and did not enter a core-EF model. Wang et al. (2023) used an affective visual working-memory paradigm with Tai Chi movement-related content and produced a markedly larger estimate than the task-neutral studies. Because the outcome may capture near transfer or stimulus familiarity, its influence was examined explicitly rather than treated as straightforward evidence of generalized updating capacity.

Two additional RCTs did not contribute an effect to the behavioral meta-analysis. Wang et al. (2025a) reported resting EEG and fitness outcomes but no eligible objective EF task. Hao examined Stroop inhibition and fNIRS in 60 participants (30 per group), but reported intervention-group pre/post reaction-time means without the corresponding control-group means and SDs. In the consistent condition, intervention-group RT decreased from 673.41 ± 168.09 to 659.34 ± 151.06 ms; in the inconsistent condition, it decreased from 814.68 ± 167.09 to 768.32 ± 118.72 ms. The report also described higher oxygenated hemoglobin across nine channels in the consistent condition and increased CH4 oxygenation over the left DLPFC in the inconsistent condition. These data establish that Tongtong (2022) assessed inhibitory-control-related performance, but they are insufficient for an unbiased concurrent-control effect size and therefore remain qualitative (Table 3).

**Table 3:** Included RCT characteristics and role in synthesis.

Study	Design; *n*	Intervention	Main eligible findings	Role
Wang and Lyu (2024)	RCT; 70	24-form; 12 wk	Improved incongruent Stroop performance; frontal theta/alpha findings	Qualitative + overall/IC
Li et al. (2025b)	RCT; 66 males	MTCC; 8 wk	Improved inhibition; no reliable updating effect; shifting estimate; mediation of phone-addiction change	Qualitative + overall/IC/WM/CF
Li et al. (2025c)	3-arm RCT; 89	24-form; 8 wk	Improved Go/No-Go and Stroop RT; no updating effect; gains attenuated at follow-up. Go RT reflected sustained attention; Stroop RT entered IC	Qualitative + overall/IC/WM; one pair
Yuan et al. (2025)	RCT; 40 females	24-form; 8 wk	Shorter affective oddball RT; exploratory N2/P3 findings	Emotional-attentional/ERP narrative only; not core EF meta-analysis
Wang et al. (2025a)	RCT; 45	24-form; 12 wk	Resting alpha and fitness outcomes; no objective EF task	Eligible neurophysiological narrative only
Tongtong (2022)	RCT; 60 (30/30)	Taijiquan; 15 wk	Within-group Stroop RT reductions and prefrontal fNIRS findings; control summary means/SDs not reported	Qualitative inhibitory-control and fNIRS narrative only; not pooled
Wang et al. (2023)	RCT; 55	Yang short form; 12 wk	Large visual 2-back effect using intervention-congruent stimuli	Qualitative + overall/WM

### 3.3 Risk of Bias assessment

Most RCTs were judged as having some concerns, principally because reporting did not always establish allocation procedures, adherence, contamination, analysis approach, or prespecified outcome selection. Participant non-blinding alone was not used as sufficient grounds for a Domain 2 judgment. DerSimonian and Laird (1986) was at high overall risk because the brief report provided insufficient information about intervention deviations and selective reporting (Table 4).

**Table 4:** Outcome-level specification for quantitative synthesis. All numerical values were cross-checked against the primary reports; positive g favors Tai Chi.

Study	Task/condition	Metric; time	Domain	Tai Chi vs control	*g* [95% CI]	Model role
Wang and Lyu (2024)	Stroop, incongruent	RT (ms); post 12 wk	IC	660 ± 70 (35) vs 710 ± 100 (35)	0.57 [0.09, 1.05]	Overall; IC
Li et al. (2025b)	Flanker: response time of inhibition	Source-reported response-time metric; post 8 wk	IC	4.20 ± 10.42 (33) vs 9.56 ± 10.38 (33)	0.51 [0.02, 1.00]	Overall; IC
Li et al. (2025b)	2-back updating	RT (ms); post 8 wk	WM	1095.57 ± 208.01 (33) vs 1136.92 ± 309.77 (33)	0.16 [−0.33, 0.64]	WM primary selected-metric model
Li et al. (2025b)	2-back updating	Correct rate (proportion); post 8 wk	WM	0.86 ± 0.12 (33) vs 0.80 ± 0.31 (33)	0.25 [−0.23, 0.74]	WM correct-response model
Li et al. (2025b)	More-Odd shifting	RT (ms); post 8 wk	CF	281.48 ± 71.12 (33) vs 305.66 ± 93.95 (33)	0.29 [−0.20, 0.77]	Single-study CF
Li et al. (2025c)	Stroop, source-reported overall RT	RT (ms); post 8 wk	IC	906.01 ± 187.91 (28) vs 1034.12 ± 214.81 (29)	0.63 [0.09, 1.16]	Overall; IC
Li et al. (2025c)	Digit-string updating	Correct-response score; post 8 wk	WM	134.39 ± 18.14 (28) vs 128.70 ± 22.51 (29)	0.27 [−0.25, 0.80]	Both WM primary selected-metric and correct-response models.
Yuan et al. (2025)	Modified affective oddball	Mean RT (ms); post 8 wk	Emotional-attentional processing	468.51 ± 27.20 (20) vs 514.06 ± 38.36 (20)	1.34 [0.65, 2.03]	Neurophysiological/behavioral narrative only; excluded from core EF pooling
Tongtong (2022)	Stroop, consistent	RT (ms); pre/post 15 wk	IC	Tai Chi: 673.41 ± 168.09 to 659.34 ± 151.06 (30); control means/SDs NR	Not estimable	Qualitative IC/fNIRS narrative only; no between-group effect
Tongtong (2022)	Stroop, inconsistent	RT (ms); pre/post 15 wk	IC	Tai Chi: 814.68 ± 167.09 to 768.32 ± 118.72 (30); control means/SDs NR	Not estimable	Qualitative IC/fNIRS narrative only; no between-group effect
Wang et al. (2023)	Affective visual 2-back	RT (ms); post 12 wk	WM	862.92 ± 65.33 (28) vs 1006.51 ± 37.11 (27)	2.65 [1.92, 3.38]	Overall; WM primary selected-metric model; task-congruent
Wang et al. (2023)	Affective visual 2-back	Accuracy rate (proportion); post 12 wk	WM	0.65 ± 0.06 (28) vs 0.48 ± 0.11 (27)	1.90 [1.26, 2.54]	WM correct-response model; task-congruent

Wang and Lyu reported reaction times in seconds; means and standard deviations were multiplied by 1,000 and are presented in milliseconds for unit consistency. This linear conversion does not change Hedges’ g. Li et al. label the Flanker measure “response time of inhibition”; because the reported values are much smaller than ordinary raw trial-level reaction times, the source label is retained without reinterpreting the variable as raw RT. Hao reported intervention-group pre/post RT means but not corresponding control-group means/SDs or an adjusted between-group estimate; no controlled effect size was calculated.

### 3.4 Quantitative Meta-analysis findings

Four RCT comparisons (*n* = 248; 124 per group) entered the overall model. Each trial contributed one effect, and no comparator was reused. The DerSimonian–Laird model yielded *g* = 1.05 (95% CI 0.23–1.87; *z* = 2.51, *p* = 0.012), with substantial heterogeneity (*q* = 27.16, df = 3, *p* < 0.001; *i*^2^ = 89.0%). This estimate should be interpreted as an average across selected task outcomes, not as a direct measure of a unitary EF construct (Table 5).

**Table 5:** RoB 2 judgments. D1, randomization; D2, deviations; D3, missing data; D4, outcome measurement; D5, selection of reported result.

Study	D1	D2	D3	D4	D5	Overall
Wang and Lyu (2024)	Some concerns	Some concerns	Low	Some concerns	Some concerns	Some concerns
Li et al. (2025b)	Low	Low	Low	Low	Low	Low risk
Li et al. (2025c)	Low	Some concerns	Some concerns	Low	Some concerns	Some concerns
Yuan et al. (2025)	Low	Some concerns	Some concerns	Some concerns	Some concerns	Some concerns
Wang et al. (2025a)	Low	Some concerns	Low	Some concerns	Some concerns	Some concerns
Tongtong (2022)	Some concerns	High risk	Low	Some concerns	High risk	High risk
Wang et al. (2023)	Low	Low	Low	Low	Some concerns	Some concerns

Substituting working-memory effects for inhibitory-control effects in the two multi-outcome trials gave *g* = 0.88 (95% CI − 0.04 to 1.80; *p* = 0.062; *i*^2^ = 91.4%). REML estimation of the primary overall model gave *g* = 1.06 (95% CI 0.08–2.05). Hartung–Knapp–Sidik–Jonkman inference for the DL model included the null (95% CI − 0.56 to 2.67; *p* = 0.130), showing that statistical significance of the broad overall model was not robust to small-sample inference. Leave-one-out estimates remained positive but varied materially, illustrating instability in the small evidence base.

The contrast between the conventional DerSimonian–Laird interval and Hartung–Knapp interval is scientifically consequential. The former suggests statistical significance, whereas the latter—designed to reflect uncertainty in estimating the random-effects mean with only four studies—produces a much wider interval crossing zero. The appropriate interpretation is not that one method is correct and the other incorrect, but that the evidence is sensitive to reasonable analytic choices. This sensitivity, together with *i*^2^ above 80%, argues against presenting the overall estimate as a stable measure of general EF enhancement.

The outcome-selection sensitivity model remained positive, but its heterogeneity was even greater. It therefore shows that the direction of the broad result is not created solely by prioritizing inhibitory-control outcomes; it does not show that dependence is irrelevant or that the effect magnitude is robust. Because the same participants contribute different candidate outcomes across alternative models, the sensitivity analyses should be read as competing specifications rather than independent confirmations.

### 3.5 EF subdomains

Inhibitory control: Three RCTs (*n* = 193) favored Tai Chi under the primary DL model (*g* = 0.57, 95% CI 0.28–0.85; *p* < 0.001; *q* = 0.10, df = 2; *i*^2^ = 0%). The low heterogeneity estimate is imprecise with only three studies and should not be interpreted as proof of a common effect (Table 6).

**Table 6:** Meta-analytic summary.

Outcome	*k*; *n*	*g* [95% CI]	*p*	*i* ^2^	Interpretation
Overall selected-task model	4; 248	1.05 [0.23, 1.87]	0.012	89.0%	Positive but highly heterogeneous and task-selection dependent
Inhibitory control	3; 193	0.57 [0.28, 0.85]	<0.001	0.0%	Most consistent evidence
Working memory: primary selected metric	3; 178	1.00 [−0.37, 2.37]	0.152	94.3%	RT prioritized; digit-string score used where RT unavailable; inconclusive
Working memory: correct responses	3; 178	0.79 [−0.19, 1.77]	0.113	89.6%	Inconclusive; same task-congruence pattern
Cognitive flexibility	1; 66	0.29 [−0.20, 0.77]	0.246	NA	Single trial; not pooled
WM primary selected metric excluding congruent task	2; 123	0.21 [−0.15, 0.56]	0.246	0%	Small, nonsignificant
WM correct responses excluding congruent task	2; 123	0.26 [−0.09, 0.62]	0.148	0%	Small, nonsignificant
Outcome-selection sensitivity	4; 248	0.88 [−0.04, 1.80]	0.062	91.4%	Positive but unstable

Working-memory primary selected metric: Three RCTs (N = 178) did not show a statistically significant pooled effect (*g* = 1.00, 95% CI − 0.37 to 2.37; *p* = 0.152; *i*^2^ = 94.3%). Excluding the intervention-congruent visual 2-back trial reduced the estimate to *g* = 0.21 (95% CI − 0.15 to 0.56; *p* = 0.246; *i*^2^ = 0%).

Working-memory correct responses/accuracy: A secondary metric-sensitivity analysis used the correct-response outcome from each of the same three trials: Li et al. 2-back correct rate (*g* = 0.25, 95% CI − 0.23 to 0.74), Sterne et al. (2019) digit-string correct-response score (*g* = 0.27, 95% CI − 0.25 to 0.80), and Wang et al. visual 2-back accuracy (*g* = 1.90, 95% CI 1.26 to 2.54). The pooled effect was not statistically significant (*k* = 3, *n* = 178; *g* = 0.79, 95% CI − 0.19 to 1.77; *z* = 1.59, *p* = 0.113), with very substantial heterogeneity (*q* = 19.23, df = 2, *p* < 0.001; *i*^2^ = 89.6%; *τ*^2^ = 0.67). Excluding the intervention-congruent Wang trial left a small, nonsignificant effect (*k* = 2, *n* = 123; *g* = 0.26, 95% CI − 0.09 to 0.62; *z* = 1.45, *p* = 0.148; *i*^2^ = 0%). The accuracy analysis therefore reproduced the qualitative pattern of the primary selected-metric model: the pooled magnitude and heterogeneity were driven by the task-congruent trial, while task-neutral performance showed no reliable benefit. Because the task-neutral primary-metric and correct-response results were both nonsignificant, the available aggregate data do not suggest an obvious speed–accuracy trade-off; however, the use of the same digit-string score in both models and the absence of trial-level speed–accuracy correlations prevent a definitive test (Figure 4).

**Figure 4:**
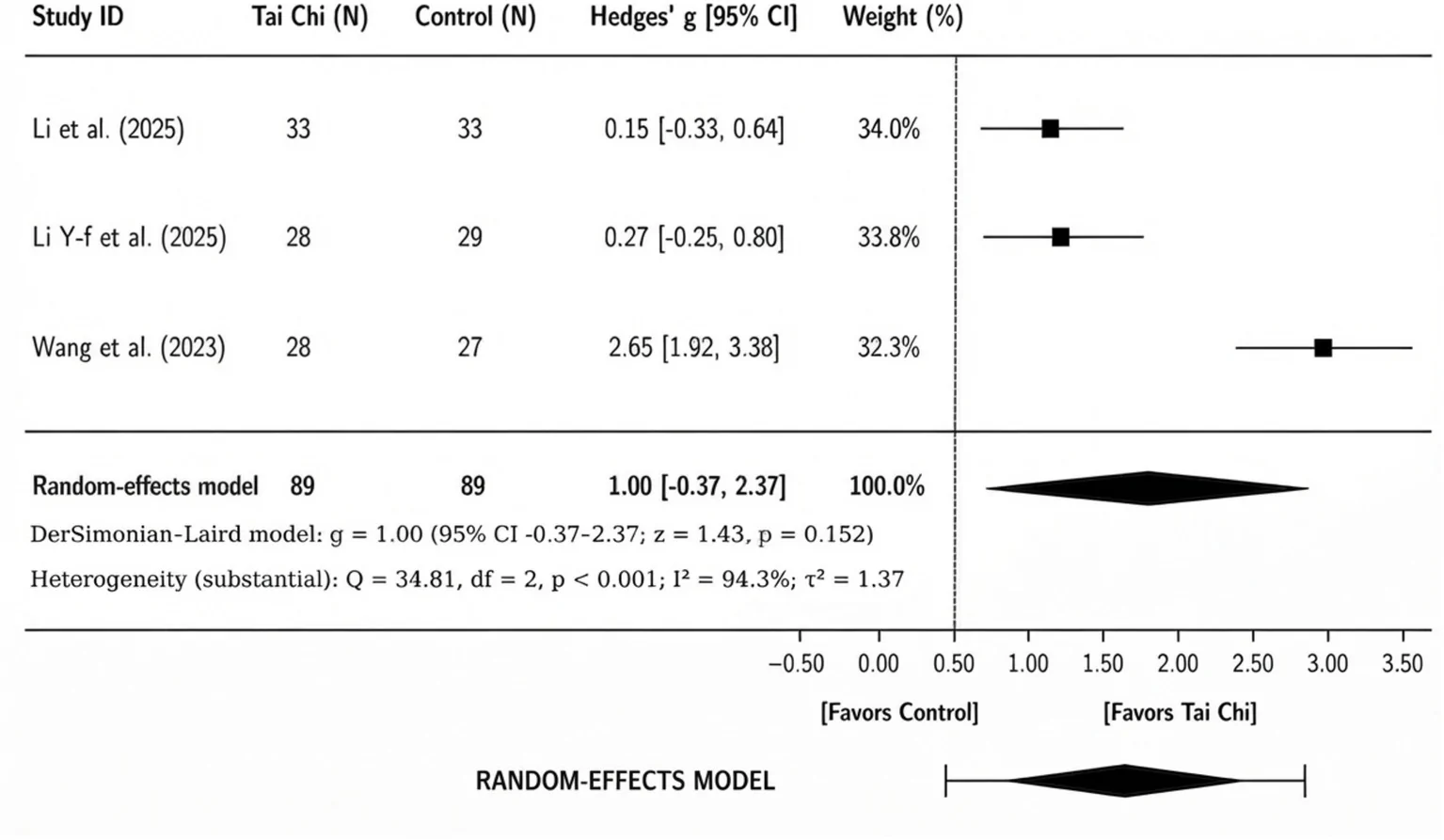
Working-memory-updating primary selected-metric model.

Cognitive flexibility: Only Li et al. (2025b) provided an eligible extractable estimate. The single-trial effect was *g* = 0.29 (95% CI − 0.20 to 0.77; *n* = 66; *p* = 0.246). No pooled effect or heterogeneity statistic was calculated.

No formal independent-effects comparison between inhibitory control and working memory was interpreted because two trials contributed to both domain models and the within-study covariance was unavailable. With only four unique contributing trials, robust moderator inference would be unreliable. Differences in point estimates are therefore descriptive only and do not establish domain-specific superiority (Figure 5).

**Figure 5:**
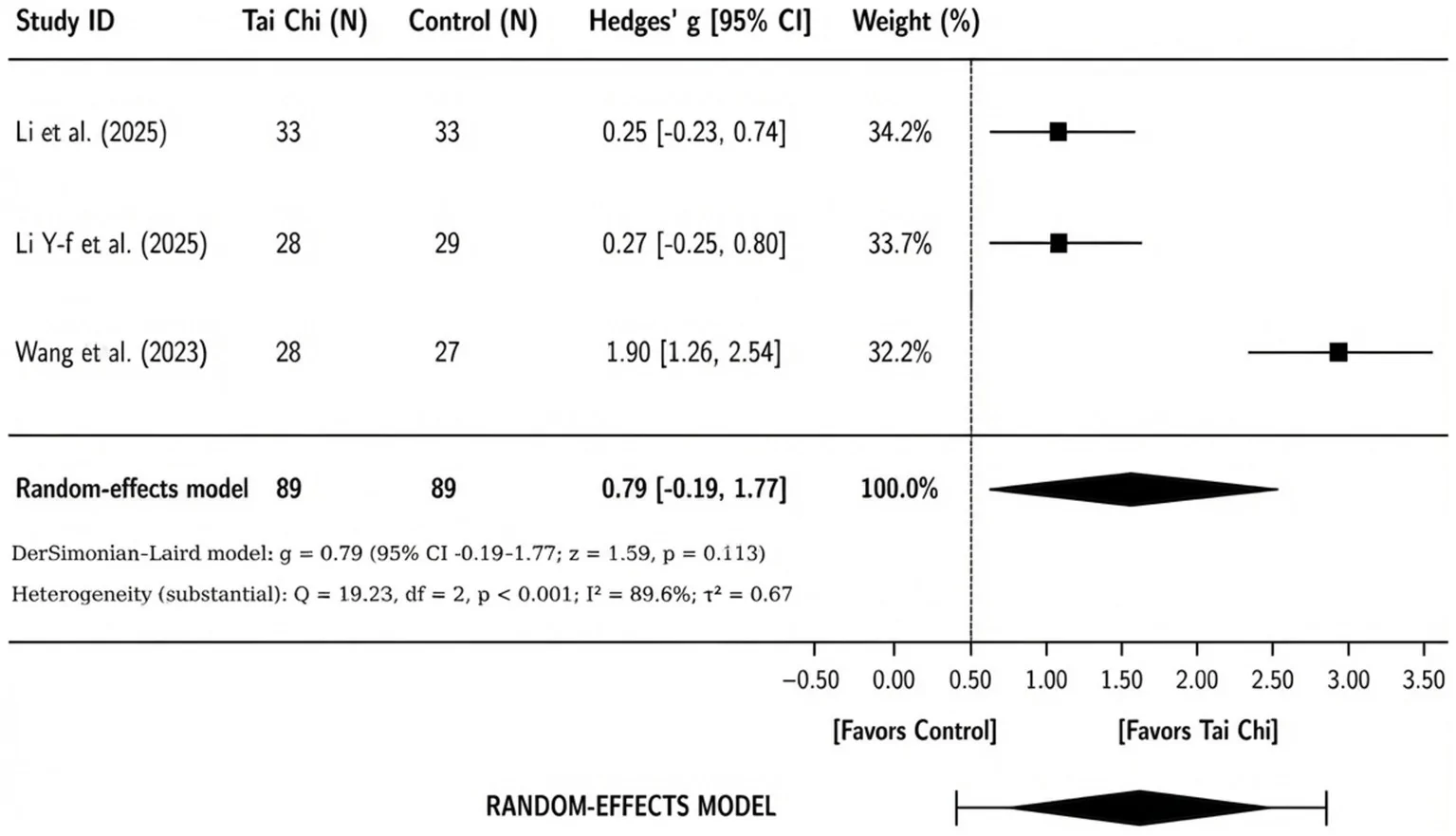
Working-memory correct-response/accuracy model.

### 3.6 Task-congruence sensitivity analysis

Removal of Wang et al. (2023), which used visual stimuli closely aligned with the trained Tai Chi movement content, reduced heterogeneity. The remaining three studies yielded *g* = 0.57 (95% CI 0.28–0.85; *i*^2^ = 0%). Importantly, all three remaining comparisons assessed inhibitory control. This analysis therefore does not independently confirm a broad task-neutral effect across EF domains; it is numerically identical to the three-study inhibitory-control model.

### 3.7 Neurophysiological and secondary outcomes

Four small heterogeneous studies reported prespecified neurophysiological outcomes. The modalities and parameters were not sufficiently comparable for pooling. Some findings were based on within-group analyses. They are therefore reported as exploratory correlates and not as established mechanisms (Table 7).

**Table 7:** Narrative neurophysiological synthesis.

Study	Modality	Finding	Interpretation
Tongtong (2022)	fNIRS + Stroop	Nine-channel oxy-Hb finding in consistent condition; increased left-DLPFC CH4 oxy-Hb in inconsistent condition; intervention-group RT decreased but control summary data NR	Qualitative inhibitory-control/hemodynamic evidence; no extractable controlled effect
Yuan et al. (2025)	ERP	Lower N2 amplitude/latency and higher P3 amplitude	Single-study electrophysiological correlate
Wang and Lyu (2024)	Resting EEG	Frontal theta change; alpha preservation	Exploratory oscillatory correlate
Wang et al. (2025a)	Resting EEG	Alpha maintained relative to control attenuation	No accompanying objective EF task

Tongtong (2022) reported that, during the consistent Stroop condition, oxygenated hemoglobin across nine channels was higher in the Tai Chi group than in control and changed from pre- to post-test (source-reported *t* = 3.28, *p* = 0.037), although channel-level means, SDs, and a clearly specified between-group contrast were unavailable. During the inconsistent condition, CH4 oxygenation over the left DLPFC increased (*t* = 3.509, *p* = 0.004). Intervention-group RT decreased from 673.41 ± 168.09 to 659.34 ± 151.06 ms in the consistent condition and from 814.68 ± 167.09 to 768.32 ± 118.72 ms in the inconsistent condition; the report gave no corresponding control means/SDs. It reported no significant control-group pre/post change (consistent *t* = 1.048, *p* = 0.339; inconsistent *t* = 0.906, *p* = 0.513). Some reported t and p combinations appear internally inconsistent under ordinary paired-t assumptions, so the values are reproduced as source-reported and not reinterpreted. Yuan et al. (2025) reported reduced N2 amplitude, shorter N2 latency to negative stimuli, and increased P3 amplitude after 8 weeks. Wang and Lyu (2024) reported frontal theta changes and preservation of alpha power, whereas Wang et al. (2025a) reported maintained resting alpha power relative to control attenuation. These isolated results require independent replication and cannot demonstrate that a neural change mediated a behavioral effect.

Li et al. (2025b) reported that change in inhibitory control statistically mediated 40.28% of the reduction in mobile-phone-addiction scores. Given the single study and self-reported behavioral outcome, this is preliminary evidence of potential transfer rather than definitive proof of real-world causal mediation. No objective academic outcome was reported.

Interpretation of fNIRS requires attention to contrast and direction. Higher oxygenated hemoglobin during task performance may reflect stronger recruitment of prefrontal resources, but it is not synonymous with efficiency. To support an efficiency interpretation, one would generally expect equal or better performance with reduced resource expenditure, or a clearly specified pattern across task difficulty. The available report instead provides a localized within-group increase and limited comparative behavioral information; the finding is therefore best described as altered hemodynamic recruitment.

ERP components similarly have context-dependent meanings. N2 is often associated with conflict detection, response inhibition, or emotional salience, while P3 relates to attentional allocation and context updating. Amplitude and latency can vary with task design, electrode selection, preprocessing, and trial count. A single study showing lower N2 and higher P3 cannot establish a sequence in which Tai Chi first suppresses conflict and then improves updating. It identifies a pattern that should be tested in preregistered replications with standardized preprocessing and correction for multiple comparisons.

Resting EEG is further removed from task-specific executive performance. Theta and alpha power vary with vigilance, fatigue, eyes-open versus eyes-closed recording, stress, and artifact handling. Preservation of alpha relative to control decline may be compatible with stress buffering, but it does not directly measure inhibition, updating, or shifting. Future studies should link preregistered EEG changes to behavioral change and test whether the association differs from an active exercise comparator (Table 8).

**Table 8:** GRADE certainty assessment.

Outcome	RoB	Inconsistency	Indirectness	Imprecision	Publication bias	Certainty; reasons
Overall model	Serious	Serious	Not serious	Serious	Not assessable	Very low: most studies had some concerns; *i*^2^ = 89.0%; only four small trials and CI compatible with moderate-to-very-large effects
Inhibitory control	Serious	Not serious	Not serious	Serious	Not assessable	Low: RoB concerns; risk-of-bias concerns; only three small trials and insufficient information size; magnitude remains uncertain
Working memory	Serious	Serious	Not serious	Serious	Not assessable	Very low: primary-metric *i*^2^ = 94.3% and correct-response *i*^2^ = 89.6%; three small studies; both CIs cross null and are task-congruence dependent
Cognitive flexibility	Serious	Not assessable	Not serious	Very serious	Not assessable	Very low: one small trial; CI crosses null; consistency cannot be assessed
Neurophysiological correlates	Serious	Serious	Serious	Very serious	Not assessable	Very low: isolated small studies, heterogeneous modalities and parameters, some within-group findings, surrogate outcomes, and no quantitative synthesis

### 3.8 Publication bias and certainty

Formal funnel-plot asymmetry tests and trim-and-fill were not performed because only four comparisons were available. Publication bias and small-study effects could not be assessed reliably. The possible absence of unpublished small or null studies cannot be assumed.

GRADE certainty was very low for the overall selected-task model, low for inhibitory control, and very low for working memory, cognitive flexibility, and the narrative neurophysiological correlates. Ratings reflect limitations in trial conduct/reporting, passive controls, heterogeneity, small information size, confidence intervals compatible with materially different magnitudes or with no effect, and—in the neural evidence—heterogeneous surrogate modalities and some within-group-only findings.

The GRADE judgment separates magnitude from certainty. A large point estimate does not create high-certainty evidence when it arises from a small, heterogeneous set of trials at risk of bias. For the overall model, the confidence interval excludes the null under the primary DerSimonian–Laird method but remains compatible with effects ranging from moderate to very large, and the Hartung–Knapp interval includes the null. The estimate also changes meaning when the task-congruent study is removed because the remaining model contains only inhibitory-control outcomes. These features justify very low certainty despite a favorable primary point estimate.

For inhibitory control, the direction was consistent enough to avoid downgrading for serious statistical inconsistency, but only three small passive-control trials contributed. The information size remained below that expected for a stable, adequately powered multicenter comparison, and the confidence interval allowed materially different magnitudes. A low-certainty rating therefore communicates that further rigorous trials are likely to change confidence in the estimate and may change its magnitude. Working memory was downgraded more severely because its confidence interval crossed the null and the studies showed extreme incompatibility. Cognitive flexibility received very low certainty because one trial cannot establish reproducibility or between-study consistency.

Publication bias was not downgraded as a separately quantified domain because it could not be assessed reliably, rather than because it was absent. With four comparisons, funnel asymmetry could reflect chance, heterogeneity, outcome selection, or selective publication, and formal regression would be unstable. This uncertainty reinforces cautious interpretation but should not be converted into a precise numerical adjustment. English-only searching may have excluded relevant Chinese-language studies.

GRADE also requires attention to indirectness. The participants were directly relevant to the student population, and the behavioral tasks were prespecified EF-related outcomes, so the table does not apply a formal indirectness downgrade. Nevertheless, indirectness remains important for practical recommendations: laboratory reaction-time differences are not direct measures of academic performance, everyday self-regulation, or long-term functioning. The absence of a formal downgrade for the laboratory question must not be interpreted as direct evidence for educational benefit.

## 4 Discussion

### 4.1 Principal findings

The revised audit identified four RCT comparisons suitable for the primary selected-task model. The overall model favored Tai Chi under conventional DerSimonian–Laird inference but showed substantial heterogeneity, was strongly influenced by one very large intervention-congruent working-memory estimate and was not statistically robust under Hartung–Knapp inference. Once the task-congruent study was removed, the remaining evidence consisted entirely of three inhibitory-control comparisons. Accordingly, the most defensible conclusion is that Tai Chi may improve short-term inhibitory-control-related performance in college students; evidence for a broader cross-domain EF effect remains insufficient (Li et al., 2025c; Wang et al., 2023).

The inhibitory-control estimate was moderate to large, but all pooled studies used passive controls. Effects may therefore incorporate nonspecific influences of structured activity, social contact, instructor attention, expectations, or repeated testing. No pooled evidence isolates Tai Chi-specific mind–body components from general exercise effects (Wang and Lyu, 2024; Li et al., 2025c; Wang et al., 2023).

Several explanations could produce positive post-test differences without a Tai Chi-specific cognitive mechanism. Students allocated to an organized class may expect improvement, interact regularly with peers and instructors, change sleep or other activity, and become more motivated during follow-up testing. Passive controls experience none of these features. Although computerized outcomes are less susceptible to assessor judgment than subjective scales, participant effort and speed–accuracy strategy remain responsive to expectation. Blinding is generally impossible, but expectancy can be measured and active controls can equalize attention and engagement.

Practice effects are another consideration. Repeated exposure to Stroop, Flanker, Oddball, or n-back tasks generally improves familiarity and response selection. Randomization should distribute ordinary retest effects across groups, yet differential engagement or baseline imbalance can modify them in small samples. Trials should use alternate stimuli where appropriate, report reliability, prespecify the primary score, and analyze group-by-time contrasts or baseline-adjusted post-test estimates rather than relying on within-group significance.

### 4.2 Subdomain evidence

Inhibitory control showed the most consistent pattern, but no valid formal domain comparison could be supported because of dependent effects and too few unique trials. Apparent differences across subdomains should not be treated as proof of selective superiority. A transfer hypothesis based on controlled movement and response suppression is plausible, but it remains theoretical (Li et al., 2025b; Wang and Lyu, 2024).

Working-memory evidence was inconclusive on both metrics. The primary selected-metric estimate was imprecise and heterogeneous (*g* = 1.00, 95% CI − 0.37 to 2.37), and the correct-response estimate showed the same pattern (*g* = 0.79, 95% CI − 0.19 to 1.77). After exclusion of the intervention-congruent trial, both task-neutral estimates were small and nonsignificant (primary selected metric *g* = 0.21; correct responses *g* = 0.26). The parallel primary selected-metric and correct-response analyses did not suggest an obvious speed–accuracy trade-off; however, a definitive test was impossible because the digit-string score was used in both models and within-person speed–accuracy covariance was unavailable. Cognitive flexibility was represented by one small trial, so the evidence is insufficient rather than evidence of no effect (Wang et al., 2023).

The inhibitory-control model is more coherent than the broad model because it is restricted to Stroop and Flanker paradigms emphasizing interference resolution. The affective oddball task was removed from this domain because it combined attentional orienting, emotional processing, and two-choice response selection without a clear prepotent-response inhibition contrast. The revised Li et al. (2025c) extraction uses source-reported Stroop reaction time because Go reaction time indexed sustained attention. Even after these corrections, the absence of condition-specific interference scores in some reports limits construct purity. The pooled estimate should therefore be described as inhibitory-control-related performance rather than a direct latent measure of inhibition (Li et al., 2025c; Yuan et al., 2025).

For working memory, the near-zero task-neutral effects in both the primary selected-metric and correct-response analyses contrast sharply with the large effects from the intervention-congruent visual 2-back task. This pattern is compatible with near transfer or stimulus familiarity rather than a generalized improvement in updating capacity. Differences in task difficulty, reliability, or participant engagement may also contribute. Future trials should administer both training-related and task-neutral updating measures, counterbalance task order, and test the intervention-by-stimulus interaction (Li et al., 2025c; Yuan et al., 2025).

The cognitive-flexibility evidence illustrates why absence of statistical significance must not be interpreted as evidence of absence. One trial with 66 participants provides a broad confidence interval compatible with a small disadvantage, no effect, or a moderate benefit. There is no between-study replication and no estimate of heterogeneity. The appropriate conclusion is that the domain remains insufficiently studied, not that Tai Chi fails to influence flexibility (Li et al., 2025b).

### 4.3 Exploratory neurophysiological evidence

A small number of heterogeneous studies reported fNIRS, ERP, and resting-state EEG changes. Yuan et al. was retained here because its affective oddball task and ERP outcomes inform emotional-attentional processing, but its behavioral RT was not treated as a core EF effect. These findings were not quantitatively pooled, were not consistently based on extractable between-group analyses and have not been independently replicated. They should be regarded as exploratory correlates rather than evidence of mechanisms. The observed ERP pattern, for example, has been interpreted as potentially reflecting differences in conflict monitoring and attentional allocation, but that interpretation rests on a single small study. Similarly, increased prefrontal oxygenation may reflect altered resource recruitment rather than greater neural efficiency; the current evidence cannot distinguish these explanations (Wang and Lyu, 2024; Wang et al., 2025a).

### 4.4 Heterogeneity and task congruence

Differences in Tai Chi style, duration, control condition, task, and score type limit interpretation of a single pooled SMD. The largest estimate came from a visual 2-back paradigm aligned with trained content. Its removal reduced heterogeneity, but it also left only inhibition studies. Future trials should distinguish near transfer on training-related stimuli from far transfer on standardized task-neutral batteries (Li et al., 2025c; Yuan et al., 2025; Wang et al., 2023).

Intervention dose could not be examined statistically. An eight-week course and a 15-week course differ not only in exposure but potentially in form mastery, instructor feedback, adherence, and participant selection. Style labels also provide incomplete information: two nominally similar 24-form programs may differ in pace, emphasis on breathing, group size, home practice, and instructor competence. Future reports should use the Consensus on Exercise Reporting Template or an equivalent framework, describe progression and fidelity, and report attendance and adverse events by group (Yuan et al., 2025; Wang et al., 2023).

Standardized mean differences permit combination of outcomes measured on different scales, but they are influenced by within-study standard deviation. Restricted, unusually homogeneous samples can yield large SMDs even when raw differences are modest, while unreliable outcomes can inflate variance and attenuate SMDs. Interpreting g as a universal cognitive unit is therefore inappropriate. Raw metrics, task reliability, and the practical meaning of response-time differences should accompany standardized effects (Li et al., 2025c).

### 4.5 Real-world and academic transfer

One trial reported mediation of mobile-phone-addiction change through inhibitory control, offering preliminary evidence of potential behavioral transfer. No study measured objective GPA, examination performance, course completion, or retention. Laboratory EF changes cannot be assumed to improve academic achievement without direct evidence (Li et al., 2025b).

Mediation analysis is especially demanding in a small intervention trial. A statistically significant indirect path depends on correct temporal ordering, reliable measurement, absence of unmeasured mediator–outcome confounding, and adequate power. When mediator and outcome changes are assessed over the same short interval, temporal precedence may be unclear. The reported mediation percentage is therefore hypothesis-generating and should not be interpreted as proof that improved inhibition caused reduced phone use (Li et al., 2025b).

Academic performance is influenced by prior achievement, course difficulty, socioeconomic conditions, motivation, attendance, mental health, sleep, and assessment practices. Even a genuine improvement in laboratory inhibition may be too small, too transient, or too context-specific to alter grades. Conversely, Tai Chi could improve wellbeing or study routines without changing laboratory EF. Future trials should prespecify academic outcomes, obtain administrative rather than self-reported records, adjust for baseline attainment, and distinguish immediate cognitive transfer from end-of-semester educational outcomes (Guo et al., 2022; Li et al., 2025a).

### 4.6 External validity

Six of seven included studies were conducted in China and one in Thailand. Participants were mostly young undergraduates, and some studies enrolled single-sex samples. Generalizability outside East and Southeast Asian tertiary settings is therefore limited.

Cultural familiarity may affect recruitment, expectations, adherence, and the meaning attributed to Tai Chi. In settings where Tai Chi is familiar and institutionally supported, students may view it as credible and socially acceptable. In less familiar settings, novelty could either increase engagement or reduce adherence. Educational schedules, baseline physical activity, and campus resources also vary internationally. Replication should therefore include culturally diverse institutions and document prior exposure, treatment preference, and expectancy (Yuan et al., 2025).

Sex-specific samples further narrow inference. A male-only trial focused on problematic mobile-phone use, while a female-only ERP trial examined emotional processing. Their findings cannot automatically be generalized across sexes or to unselected student populations. Future studies should recruit adequately diverse samples, report sex and gender clearly, and avoid underpowered subgroup claims unless interaction tests are prespecified.

### 4.7 Implications for research and practice

The current evidence does not justify recommending Tai Chi as a direct academic-performance intervention. It may nevertheless be a reasonable voluntary physical-activity option because it is generally low impact, adaptable to limited space, and may support wellbeing. Any institutional implementation should be justified primarily as a health or recreational program rather than as a proven cognitive-enhancement strategy. Claims about grades, neural efficiency, or domain-general executive improvement should be avoided.

A definitive trial would use concealed randomization, blinded outcome assessors where feasible, preregistered outcomes and analysis, and an active comparator matched for duration, social contact, instructor attention, expectancy, and exercise intensity. A three-arm design comparing Tai Chi, intensity-matched aerobic exercise, and wait-list control could separate Tai Chi-specific effects from general activity and participation effects. Sample-size calculations should be based on a conservative effect rather than the current small-study pooled estimate (Li et al., 2025b; Wang and Lyu, 2024; Li et al., 2025c; Yuan et al., 2025; Wang et al., 2023).

The cognitive battery should include at least one validated task-neutral measure for each EF subdomain, with a single declared primary outcome or a prespecified composite. Accuracy and reaction time should be interpreted jointly to detect speed–accuracy trade-offs. Reliability, exclusion rules, trial counts, preprocessing, and scoring formulas should be reported. If training-related stimuli are included, they should be analyzed as near-transfer outcomes and contrasted formally with neutral stimuli (Diamond, 2013; Miyake et al., 2000).

Mechanistic studies should be embedded within adequately powered behavioral trials rather than conducted as isolated small experiments. Neurophysiological hypotheses, channels/electrodes, frequency bands, time windows, preprocessing, and multiplicity correction should be preregistered. Between-group changes should be reported with effect estimates and confidence intervals, and mediation should be attempted only when temporal ordering and sample size are adequate. Long-term follow-up should determine whether continued practice is necessary to maintain any benefit (Li et al., 2025b; Wang and Lyu, 2024; Li et al., 2025c; Yuan et al., 2025; Wang et al., 2023).

Finally, objective academic and functional outcomes should be measured directly. Administrative GPA, examination scores, attendance, assignment completion, and retention can be linked to cognitive and wellbeing outcomes while preserving confidentiality. Such trials should specify whether academic outcomes are primary, secondary, or exploratory and should account for baseline performance and course clustering.

## 5 Limitations

1. Only four RCT comparisons (*n* = 248) could be pooled in the primary selected-task model. Subdomain analyses were small; both working-memory metric models contained only three trials, and cognitive flexibility had only one eligible estimate.
2. All four primary pooled comparisons used passive or routine-activity controls, preventing separation of Tai Chi-specific effects from nonspecific exercise, social, attention, and expectancy effects.
3. Tai Chi style, dose, delivery, task, and outcome metric varied substantially. The overall model summarized selected standardized outcomes and should not be interpreted as a direct measure of a single unitary EF construct.
4. The review was not prospectively registered. Outcome-selection rules were made explicit during revision but were not documented in a prospective public protocol. Registration of a future update does not correct this limitation.
5. The evidence was geographically concentrated, predominantly in China, and long-term follow-up was rare.
6. Neurophysiological findings came from a few heterogeneous studies, sometimes using within-group analyses, and were not suitable for meta-analysis. Hao did not report control-group means/SDs for Stroop outcomes, and several source-reported t and p combinations could not be reconciled under ordinary paired-t assumptions.
7. Publication bias could not be assessed reliably with four comparisons.
8. The search was restricted to English-language records, potentially introducing language bias and excluding eligible non-English evidence.

An additional limitation concerns outcome construct validity. Several primary reports used broad task labels without fully reporting condition-specific scoring, and one source classified Go reaction time as sustained attention. Although the revised audit corrected the pooled assignment, incomplete task reporting limits certainty that every estimate isolates the intended EF component. The standardized scale cannot correct construct misclassification.

The review also depends on aggregate published data. Individual-participant data were unavailable, preventing consistent baseline adjustment, verification of distributional assumptions, analysis of speed–accuracy trade-offs, and exploration of effect modification by sex, baseline performance, stress, or adherence. Small samples may produce non-normal reaction-time distributions, yet transformed or robust estimates were generally unavailable.

## 6 Conclusion

Tai Chi may improve short-term inhibitory-control performance in college students (*g* = 0.57, 95% CI 0.28–0.85), but the certainty of this evidence is low and only three trials contributed. Evidence for working-memory updating is inconsistent on both primary selected metric (*g* = 1.00, 95% CI − 0.37 to 2.37) and correct responses/accuracy (*g* = 0.79, 95% CI − 0.19 to 1.77), and both models are strongly influenced by one unusually large, task-congruent estimate; cognitive flexibility is represented by only one eligible trial. Because all pooled comparisons used passive controls, the observed effects cannot be separated from nonspecific effects of exercise participation, social interaction, instructor attention, or expectancy. Separate small studies reported exploratory fNIRS, ERP, and EEG changes, but these findings do not establish neurophysiological mechanisms. Larger preregistered RCTs using active comparators, task-neutral cognitive batteries, long-term follow-up, and objective academic outcomes are required.

## Appendix A Complete search strategies

Searches covered database inception to 15 April 2026, were restricted to English-language records, and had no date or publication-status restrictions.

*PubMed*: [(Tai Ji [Mesh] OR Tai Chi [tiab] OR Taijiquan[tiab]) AND (Students [Mesh] OR Young Adult [Mesh] OR university student* [tiab] OR undergraduate*[tiab] OR postgraduate*[tiab] OR young adult* [tiab]) AND (Executive Function [Mesh] OR Memory, Working [Mesh] OR executive function* [tiab] OR inhibitory control [tiab] OR working memory [tiab] OR cognitive flexibility [tiab] OR Stroop[tiab] OR Flanker[tiab] OR Go/No-Go [tiab] OR n-back [tiab] OR EEG[tiab] OR ERP[tiab] OR fNIRS[tiab])].

*Web of Science Core Collection*: TS = [(“Tai Chi” OR Taijiquan OR “Tai Chi Chuan”) AND (“university student*” OR undergraduate* OR postgraduate* OR “young adult*” OR “college student*”) AND (“executive function*” OR “executive control” OR “inhibitory control” OR “working memory” OR “cognitive flexibility” OR Stroop OR Flanker OR “Go/No-Go” OR “n-back” OR EEG OR ERP OR fNIRS OR “cognitive control”)].

*Scopus*: TITLE-ABS-KEY [(“Tai Chi” OR taijiquan) AND (“university student*” OR undergraduate* OR postgraduate* OR “young adult*”) AND (“executive function*” OR “inhibitory control” OR “working memory” OR “cognitive flexibility” OR stroop OR flanker OR “Go/No-Go” OR “n-back” OR eeg OR erp OR fnirs)].

*CNKI*: (TI = ‘young adult’ OR AB = ‘young adult’) AND (TI = ‘executive function’ OR AB = ‘executive function’ OR TI = ‘inhibitory control’ OR AB = ‘inhibitory control’ OR TI = ‘working memory’ OR AB = ‘working memory’ OR TI = ‘cognitive flexibility’ OR AB = ‘cognitive flexibility’ OR TI = ‘Stroop’ OR AB = ‘Stroop’ OR TI = ‘Flanker’ OR AB = ‘Flanker’ OR TI = ‘Go/No-Go’ OR AB = ‘Go/No-Go’ OR TI = ‘n-back’ OR AB = ‘n-back’ OR TI = ‘EEG’ OR AB = ‘EEG’ OR TI = ‘ERP’ OR AB = ‘ERP’ OR TI = ‘fNIRS’ OR AB = ‘fNIRS’).
